# Clinical and polysomnographic findings in class III obese patients

**DOI:** 10.1016/S1808-8694(15)30606-6

**Published:** 2015-10-18

**Authors:** Rodrigo de Paiva Tangerina, Fernanda Louise Martinho, Sonia Maria Togeiro, Luiz Carlos Gregório, Sergio Tufik, Lia Rita Bittencourt

**Affiliations:** 1 Graduate student.; 2 Master's degree student.; 3 Doctor, faculty member of the Psychobiology Department, UNIFESP-EPM.; 4 Doctor, faculty member of the Otorhinolaryngology and Head & Neck Surgery Department, UNIFESP-EPM.; 5 Livre-docente habilitation, full professor of the Psychobiology Department, UNIFESP-EPM.; 6 Doctor, faculty member of the Psychobiology Department, UNIFESP-EPM. Otorhinolaryngology and Head & Neck Surgery Department, UNIFESP-EPM e Psychobiology Department, UNIFESP-EPM.

**Keywords:** morbid, obstructive, obesity, sleep apnea

## Abstract

The Obstructive Sleep Apnea/Hipopnea Syndrome (OSAHS) is closely related to obesity; a linear relation, however, has not been established, particularly in morbid obesity patients.

**Aim:**

To evaluate clinical and polysomnographic findings in a group of class III obese patients, and to relate these findings with the presence or absence of OSAHS.

**Material and Method:**

Forty five patients with body mass indexex (BMI) over 40Kg/m2 were selected consecutively. A clinical history, the anthropometric examination and polysomnography were undertaken in all patients. The results of a study group and a control group (with no OSAHS) were compared.

**Results:**

The sample consisted of 68.9% female and 31.1% male patients. The average age was 46.5 years (SD - 10.8 years); the average BMI was 49 (SD - 7 Kg/m2) and the average neck circumference was 43.4 cm (SD - 5.1 cm). All subjects were habitual snorers and 48.9% had daytime drowsiness. Polysomnography showed that 77.8% had an apnea/hipopnea index over 5. The findings associated with OSAHS were: younger age (p=0,02) and an increased neck circumference (p=0.004).

**Conclusion:**

The prevalence of OSAHS was very high, which emphasizes the importance of investigating this syndrome in patients sent for bariatric surgery. The neck circumference was the best OSAHS marked in this group of patients.

## INTRODUCTION

The obstructive sleep apnea hypopnea syndrome (OSAHS) is currently defined according to the American Academy of Sleep Medicine (AASM) classification published in 2005.^1^ Since the first published papers on OSAHS, this condition has always been associated with excess weight,^2^, ^3^ being more prevalent in middle aged obese individuals. OSAHS affects about 4% of males and 2% of females in the general population.4 In 1999, an AASM task force graded OSAHS based on medical and polysomnographic findings.^5^ The apnea-hypopnea index (AHI) was used as the polysomnographic variable; it is the number of apnea and hypopnea events for each hour of sleep. OSAHS may be classified as mild when the AHI is between 5 and 15; it is moderate between 15 and 30, and severe when above 30. OSAHS may also be related with various other conditions such as systemic arterial hypertension, acute myocardial infarction, strokes, and traffic accidents, among others. It has thus become a public health issue.^6^, ^7^, ^8^, ^9^ Obesity itself is fast becoming a pandemic.10 The World Health Organization classifies obesity based on the body-mass index (BMI), which is weight (in kilograms) divided by the square of the height (in meters). A person is eutrophic when this index is between 18.5 and 24.9 Kg/m². Overweight is when the BMI is between 25 and 29,9. Class I obesity is when the BMI is between 30 and 34.9. Class II obesity is when the BMI is between 35 and 39.9. A BMI of 40 or above defines class III obesity, which has been named morbid obesity.^11^ Class III obesity patients are at a high risk for comorbidities;^11^ this becomes even more relevant when we note that these same comorbidities are present in OSAHS.^12^ The purpose of this paper was to describe the medical and polysomnographic features of a group of class III obesity patients.

## MATERIAL AND METHOD

This study included 45 consecutive patients from the obesity clinic of the Gastric Surgery Discipline, seen between June 2005 and January 2006, chosen according to inclusion and exclusion criteria, who had been referred to bariatric surgery. Inclusion criteria were: adults of both sexes aged between 18 and 75 years, with a BMI equal to or over 40 Kg/m². Exclusion criteria were: having undergone surgery or medical therapy for snoring or OSAHS, more than 10% variation of body weight (gain or loss) between the dates of polysomnography and the physical examination, and using sedative or stimulant drugs. A guided clinical history was taken, and anthropometric measurements were done as follows: height, weight and neck circumference. Baseline polysomnography was done in a specialized clinic. A descriptive analysis of this population was made, as well as a comparative analysis between patients with and with no OSAHS (Student's t test and the X²). The Research Ethics Committee of the institution approved this paper (protocol number 1223/05).

## RESULTS

There were 45 patients, 14 male (31.1%) and 31 females (68.9%). Age ranged from 22 to 74 years (mean 46.5 ±10.8 years). The BMI ranged from 40 to 71 kg/m² (mean 49 ±7 kg/m²). The AHI throughout the group ranged from zero to 109.6 events per hour (mean 30.8 ±31.9 events per sleeping hour). The neck circumference ranged from 33 to 58 cm (mean 43.4 ±5.1cm). All patients were habitual snorers and 48.9% complained of excessive daytime drowsiness.

Ten subjects had an AHI below five events per hour (22.2%) and 35 subjects had an AHI above five events per hour (77.8%). The former group (non-apneic) consisted only of female subjects, and the latter group (apneic) contained 14 male subjects (40%) and 21 female subjects (60%). Age ranged from 44 to 74 years (mean 53.1 ±9.1 years) in the non-apneic group. The BMI ranged from 41.9 to 57.7 Kg/m² (mean 47.8 ±6 Kg/m²). The AHI ranged from zero to five events per hour (general mean 2.96 ±1.6 events per sleeping hour). The neck circumference ranged from 37 to 42cm (mean 39.5 ±2cm). In the group of apneic subjects, age ranged from 22 to 61 years (mean 44.6±10.6 years). The BMI ranged from 40 to 71 Kg/m² (mean 49.8±6,.9 Kg/m²). The AHI ranged from 6.5 to 109.6 events per hour (general mean 38.7±32 events per sleeping hour). Of these patients, 34.3% had mild apnea (AHI between five and 15), 25.7% had moderate apnea (AHI between 15 and 30), and 40% had severe apnea (AHI over 30 events per sleeping hour). The neck circumference ranged from 33 to 58 cm (mean 44.6±5.2cm). A comparison of the clinical features in both groups may be found in Table 1; a comparison between the polysomnographic features is shown in Table 2.

**Table 1:** Clinical features of both groups.

	Non-Apneic (n=10)	Apneic (n=35)	p value
Agee (years)	53,1 ±9,1	44,6 ±10,6	0,02*
BMI (Kg/m2)	47,8 ±6	49,8 ±6,9	ns*
Neck circumference (cm)	39,5 ±2	44,6 ±5,2	0,004*

BMI: body mass index;

ns: not statistically significant;

* Based on Student's t testt

**Table 2:** Polysomnographic features of both groups.

	Non-Apneic (n=10)	Apneic (n=35)	p value
AHI	2,9	38,7	0,001
O2min	83	73	0,001
Sleep efficiency (%)	72	74	ns
Stage 1 (%)	6	8	ns
Stage 2 (%)	59	65	ns
Stage 3/4 (%)	15	10	ns
REM	19	16	ns

AHI: apnea hypopnea index;

O2 min: minimum oxyhemoglobin saturation;

ns: not statistically significant.

In the apneic and non-apneic groups the features related with the presence of OSAHS were: younger age (p=0.02) and a higher neck circumference (p=0.004). There was no statistically significant proportion between a higher BMI and increased severity of OSAHS.

## DISCUSSION

OSAHS was found in 77.8% of 45 subjects, showing that OSAHS is highly prevalent in class III obese patients. Its prevalence in the general population is between 2 to 4%.^4^ Our prevalence of OSAHS in this study is similar to published results.^13^ There were 35 OSAHS patients, of which 40% were male and 60% were female. This proportion is different from classically described results for OSAHS, which has shown a two to one male to female rate.^4^ This difference may be explained by the fact that our sample originated from a digestive surgery outpatient unit that aims to perform bariatric surgery; the number of women seeking this type of therapy is higher than the number of men, at times over 70% of patients that undergo this procedure.^14^

The mean age in the non-apneic group was 53.1±9.1 years; the mean age in the apneic group was 44.6±10.2. There was a statistically significant age difference. Curiously, the apneic group was younger than the non-apneic group. We had expected that the non-apneic group would have been younger, because of the possibility that pharyngeal tissue and muscle flaccidity - which occurs naturally with age - would be related to the pathophysiology of OSAHS. We found, however, that both groups were in the age group with maximum prevalence of OSAHS,4 leading us to believe that the age difference in these groups of middle-aged adults was less relevant than other features found in the class III obese patients. Although there is no doubt about the relation between obesity and OSAHS, we found no direct correlation between the higher BMIs and AHIs. This suggests that above a certain value, additional BMI scores do not have the same impact on OSAHS as happens with eutrophic or class I obese subjects.

Snoring was found in all patients of this sample, showing that its presence should not be used as indicative of OSAHS in this group. Excessive daytime drowsiness was reported by 48.9% of subjects. As OSAHS was detected in 77.8% of cases, this complaint isolatedly is not a good indicator of this condition; the risk in doing so is to underestimate the diagnosis.

The analysis of polysomnography showed that a high AHI in OSAHS patients affects mostly oxyhemoglobin saturation; the minimum oxyhemoglobin saturation was significantly lower in apneic patients. This effect was not seen in the sleep architecture of these patients; there was no statistically significant difference in sleep efficiency or in the distribution of sleep stages.

The mean neck circumference in the non-apneic group was 39.5±2cm; in the apneic group it was 44.6±5.2cm. This difference was statistically significant (p=0.004). Our analysis revealed that the neck circumference was related both with the presence and the severity of OSAHS. In the literature, the neck circumference has been described as one of the best independent predictive variables of OSAHS.^15^, ^16^

## CONCLUSION

The prevalence of OSAHS in this sample with class III obesity was high, confirming its existing correlation with obesity. This condition should be investigated in patients that are referred to bariatric surgery units. The neck circumference was the main marker for the presence of OSAHS in this sample.
